# Supplementary material for: The insect- and plant-associated lifestyles of Pseudomonas protegens CHA0 are preserved following serial passage through insect larvae
Source: PLoS Pathog. 2026 Sep 21;22(9):e1014101. doi: 10.1371/journal.ppat.1014101 (PMC13592716; doi:10.1371/journal.ppat.1014101)
Supplement: Supplementary file 1 [file ppat.1014101.s001.pdf]

Supplementary Information

Contents

Methods 2

- DNA isolation 2
- Hackflex library preparation 2
- Enrichment analysis 2
- Analysis of reads not mapping to CHA0 3
- *Pseudomonas* inoculums preparation for bioassays 3
- Insect virulence: *Plutella xylostella* feeding assay 3
- Plant protection: *Pythium ultimum*-cucumber assay 4
- Fungal inhibition assays 5
- Isolation of *P. xylostella* gut isolates 5
- Bacterial inhibition assays 6
- Swarming assay 6
- Biofilm formation assay 7
- *In vitro* growth assay 7

Bibliography 8

Methods

DNA isolation

Genomic DNA was isolated using the QIAamp DNA Mini Kit (Qiagen, Venlo, The Netherlands; Ref: 51306). Shortly, thawed bacterial pellets were mixed with 160 µl buffer ATL and 20 µl proteinase K, vortexed and incubated at 56°C and 300 rpm for 1 h. To remove RNA, 4 µl RNase A (Qiagen, Venlo, The Netherlands; Ref: 1007885) were added, carefully mixed with the lysed sample and incubated at room temperature for 5 min. Subsequently, the manufacturer's instructions were followed from step 2 including all recommended and optional steps. DNA was eluted in buffer AE and stored at -20°C until use.

Hackflex library preparation

Paired-end DNA libraries for Illumina sequencing were prepared using the Illumina DNA Prep kit (Illumina, San Diego, CA, USA; Ref: 20060059) according to the Hackflex protocol by Gaio et al. [1]. Briefly, 100 ng gDNA in 10 µl nuclease-free water were mixed with 25 µl tagmentation buffer and 10 µl 1:50 diluted bead-linked transposase (BLT) beads. The reaction was incubated at 55°C for 15 min and then held at 10°C. Tagmentation stop buffer was warmed for 10 min at 37°C and 10 µl thereof were added to the reaction. The reaction was incubated at 37°C for 15 min and then held at 10°C. DNA attached to the magnetic beads was washed three times with washing buffer. PCR reaction (12.5 µl nuclease-free water, 22.5 µl KAPA mix (KAPA HiFi HotStart ReadyMix PCR Kit, Roche Diagnostics, Basel, Switzerland; Ref: 50-196-5299), 5 µl of each i5 and i7 primer) was mixed with the beads and DNA was amplified with the following settings: 3 min at 68°C, 3 min at 98°C, 12x (30 s at 98°C, 30 s at 62°C, 2 min at 68°C), 1 min at 68°C and ∞ at 10°C. PCR was cleaned up with AMPure XP beads (Beckman Coulter, Brea, CA, USA; Ref:A6388) following the manufacturer's instructions with the following deviations: 40 µl instead of 20 µl bead solution were mixed with 45 µl PCR product.

Enrichment analysis

All coding sequences hit by a population level mutation were re-annotated using EggNOG 5.0 [2]. The counts of each COG (Clusters of Orthologous Genes) category and each KEGG (Kyoto Encyclopedia of Genes and Genomes) pathways in the coding sequences hit by a mutation in any cycle (= genes of interest) were compared to the expected counts derived from all CHA0 coding regions using an exact binomial test in R-studio. Enrichment scores (ES, Eq 1) were calculated with the adjusted *P*-values using the Benjamini-Hochberg method to correct for multiple testing.

$$ES = -log_{10}p\text{-}value \quad (1)$$

Analysis of reads not mapping to *Pseudomonas protegens* CHA0

On average > 99% of the population-level reads could be mapped to the the reference genome of *Pseudomonas protegens* CHA0 using SNIPPY. The reads that could not be mapped were further processed to detect potential contaminants accumulating during the cycles. The reads were assigned to taxonomic labels using Kraken2 (2.1.2) [3] with the PlusPF (version: 10/15/2025) database which included bacterial, archaea, viral, plasmid, human, protozoan and fungal RefSeq sequences. The number of assigned reads was correlated with the cycle number for each line and taxonomic label. *P*-values were adjusted using the Benjamini-Hochberg method to correct for multiple testing.

Pseudomonas inoculums preparation for bioassays

Inoculums were prepared for all bioassays as follows: Ancestral CHA0 and single clones were stored in 43% glycerol at -80°C and grown on King's B (KB; 20 g proteose peptone, 8.4 ml 87% glycerol, 1.5 g $MgSO_4$ x $7H_2O$, 1.5 g K_2HPO_4 x $3H_2O$ dissolved in 1 l ddH_2O) [4] agar supplemented with 13 µg/ml chloramphenicol, 100 µg/ml cyclohex-imide, and 10 µg/ml gentamycin (KB^{++G}) for 2 days at 24°C. Overnight cultures were grown in lysogenic Broth (LB; 10 g Bacto Tryptone, 5 g Bacto Yeast Extract, 0.25 g $MgSO_4$ x $7H_2O$, 8 g NaCl dissolved in 1 l ddH_2O) at 24°C and 180 rpm prior to experiments. To avoid displacement of subpopulations through extended growing times, populations of passaged lines were directly incubated in LB using the complete frozen aliquots without prior growth on KB^{++G} agar. For plant protection and fungal inhibition assays, 200 µl aliquots of the overnight cultures were plated on KB agar and additionally grown for 24 h at 24°C. Overnight cultures were spun down whereas bacterial lawns on agar plates were scraped off. In both cases, the cell material was re-suspended in 0.9% NaCl solution (saline) (or ddH_2O for plant protection and fungal inhibition assays) and washed twice (one volume, centrifugation for 10 min at 4°C and 2700 rpm (Allegra X12R Centrifuge, Rotor: SX4750, Beckman Coulter Life Sciences)). Cell concentration was adjusted to the desired optical density at 600 nm (OD_{600}) with an OD_{600} of 0.2 approximately corresponding to $3*10^8$ colony forming units (CFUs)/ml. Saline or ddH_2O were used for the negative control treatment.

Insect virulence: *Plutella xylostella* feeding assay

All passaged lines were screened for differences in killing ability and colonisation of *Plutella xylostella* larvae. *Pseudomonas* inoculums were adjusted to an OD_{600} of 0.02

and *P. xylostella* rearing, infection and monitoring was performed as described in the main text. Colonisation of single larvae was determined following surface-disinfection and homogenisation as described in the main text. Homogenates were 10-fold serially diluted in saline and spotted on KB^{++G} agar. Plates were incubated at 24°C for 24 h and then stored at 3°C until CFU counting. Per line, 48 larvae were monitored, six of them were sacrificed at 20, 24 or 48 h post infection (hpi) depending on the killing speed in the respective experiment for CFU determination and each line was tested in three independent experiments. CFUs/larva were $\log_{10}$-transformed for further analysis and points below the detection limit (50 CFUs/larva) were set to 25 CFUs/larva.

Plant protection: *Pythium ultimum*-cucumber assay

Protection of cucumber plants against the oomycete *Pythium ultimum* Pu-11 (see Table 1) was screened in a pot assay as described by Vesga et al. [5] with the following adjustments: 200 g of a 4:1 Jiffy peat substrate (Jiffy Products International, Moerdijk, the Netherlands)/quartz sand mixture was used per pot. 0.3 g *P. ultimum*-millet mixture were added per pot (+Pu) with sterilised millet as a *P. ultimum* negative control (-Pu). The *Pseudomonas* suspensions were adjusted to OD_{600} = 0.25 and 50 ml of the respective suspension were added per pot. This resulted in a final concentration of approximately 7.5×10^7 cells/g soil. Control pots without bacteria were amended with the same amount of water. Plants were grown in green-house conditions (70% relative humidity; day: 16 h, 22°C, 15 kLux; night: 8 h, 20°C), and rotated and watered every 2-3 days. Per line, five pots each with and without *P. ultimum* were inoculated and each line was tested in two to three independent experiments.

After 11 days, shoot fresh weight (SFW) and root colonisation were assessed per pot. Based on the shoot fresh weight, percentage impact on plant growth (PG, Eq 2), disease severity (SE, Eq 3), and disease suppression (DS, Eq 4) were calculated.

$$PG = ((SFW_{bacteria}^{-Pu}/mean(SFW_{negative}^{-Pu})) - 1) * 100 \tag{2}$$

$$SE = ((SFW_{treatment}^{-Pu}/SFW_{treatment}^{+Pu}) - 1) * 100 \tag{3}$$

$$DS = ((mean(SE_{negative})/SE_{bacteria}) - 1) * 100 \tag{4}$$

To determine CFUs per g fresh root weight (CFUs/g root), roots were washed, weighed and added to 20 ml saline in 50 ml Falcon tubes. Tubes were shaken for 30 min at 300 rpm and 3°C. Root washes were 10-fold serially diluted in saline and spotted on KB^{++G} agar. Plates were incubated at 24°C for 24 h and then stored at 3°C until CFU counting. The colonies were verified as CHA0 or CHA0 derivates based on their emission of green fluorescence. CFUs/g root were log_{10}-transformed for further analysis and points below the detection limit (114 CFUs/g root) were set to 57 CFUs/g root.

Fungal inhibition assays

Fusarium oxysporum f. sp. *radicis-lycopersici* FORL22, *Colletotrichum orbiculare* D323 and *P. ultimum* Pu-11 (see Table 1) were grown on malt agar for 2 weeks at 24°C, 4 weeks at 24°C or 2 weeks at 18°C, respectively. Plugs (ø 8 mm) of fungal mycelium were placed centrally on malt agar either 4 days before (*C. orbiculare*) or on the same day as *Pseudomonas* inoculums (OD_{600} = 0.1) were re-streaked using inoculation loops (ø 3 mm) in a square around the plug at a minimal distance of 23 mm (*C. orbiculare, F. oxysporum*) or 46 mm (*P. ultimum*). Plates were incubated at 24°C (*C. orbiculare, F. oxysporum*) or 18°C (*P. ultimum*). Radial growth (RG) of the fungus was measured twice per replicate at 3 days post inoculation (dpi) (*P. ultimum*, *F. oxysporum*), 7 dpi (*C. orbiculare, F. oxysporum*) or 14 dpi (*C. orbiculare*). The incubation temperature, inoculation and measurement time points depended on the growth speed of the antagonistic fungus and aimed to measure radial growth at a time point before the fungal mycelium in the negative control reached the place where the bacterial treatment was inoculated but also at a time point when differences between the negative control and the CHA0 treatments were best visible. Per line, 5-6 replicate plates were measured and each line was tested in three independent experiments. Percentage of fungal growth inhibition (FI, Eq 5) by each *Pseudomonas* inoculum compared to the negative control was calculated:

$$FI = ((RG_{bacteria}/mean(RG_{negative})) - 1) * 100 \tag{5}$$

Recovery of *P. xylostella* gut isolates: *Arthrobacter* sp. Px40, *Microbacterium* sp. Px99

P. xylostella eggs obtained from Syngenta Crop Protection AG (Stein, Switzerland) were reared as described by Flury et al. [6] but on chinese cabbage (Sagensitz, Oberdorf, Switzerland) in a climate chamber (60% relative humidity; day: 16 h, 25°C, 12 kLux; night: 8 h, 20°C) and starved for 3-4 h prior to transfer. Pellets of artificial food were placed on wet filter papers in 128-well insect trays and one second instar larvae was

added per well. At 3 dpi, 25-30 moribund larvae were surface-disinfected, homogenised and diluted as described in the main text. Serial dilutions were plated on LB and R2-A agar and incubated for 4 days at 24°C. Single colonies were picked and regrown on KB_{++G} agar. Overnight cultures were mixed 1:1 with 87% glycerol and stored at -80°C for long-term storage. For bacterial identification, DNA was extracted from cell pellets using the Wizard Genomic DNA Purification Kit (Promega, Madison, WI, USA) according to the manufacturer's instructions, the 16S sequence was amplified with the universal primers 27F/1492R [7] and Sanger sequenced. Some of the most abundant isolates were identified as an *Arthrobacter* sp. and a *Microbacterium* sp. named Px40 and Px99 (see Table 1), respectively.

Bacterial inhibition assays

The antagonistic bacterial strains stored in 43% glycerol at -80°C were grown on LB agar (*Arthrobacter* sp. , *Microbacterium* sp.) or LB agar supplemented with 50 µg/ml kanamycin (LB^K) (*Photorhabdus laumondii* DJC, *Xenorhabdus bovienii* SM5, see Table 1) for 2 days at 24°C. Overnight cultures were grown in LB at 24°C and 180 rpm prior to experiments. Warm LB agar was mixed with the overnight cultures (100-200 µl culture per 50 ml agar) and poured in square plates. 1 µl drops of the *Pseudomonas* inoculums (OD_{600} = 0.5) were carefully added on top of the agar using reverse pipetting to avoid splashing. Treatment positions on plates were approximately 18 mm apart and each treatment was added six times. After 2 days of incubation at 24°C the inhibition zones were clearly visible with all antagonistic bacteria and differences between the negative control and the CHA0 treatments were best visible. Therefore, pictures were taken from the plates and inhibition zone sizes were measured three times per position using the line drawing tool on Fiji (ImageJ, Version: 2.14.0). Each line was tested in two to five independent experiments.

Swarming assay

The swarming ability of all lines was screened following a protocol by Sánches-Gil et al. [8] with a few adjustments. Briefly, 0.1x Cook's Cytophaga agar (CCA) with 0.4% agar (Agar, European Type, (Ingredient) for microbiology, plant tissue culture, bacteriology grade, A0949, AppliChem, Darmstadt, Germany) was poured and dried for 30 min without the lid in a sterile bench. 1 µl drops of the *Pseudomonas* inoculums (OD_{600} = 0.5) were carefully added on top of the agar using reverse pipetting to avoid splashing. Treatment positions on plates were approximately 36 mm apart and each treatment was added six times. Plates were incubated for 18 h at 24°C. Pictures were taken from the plates and the swarm diameter was measured twice per position using the line drawing tool on Fiji. Each line was tested in two independent experiments.

Biofilm formation assay

Pseudomonas inoculums (OD_{600} = 0.5) were first diluted 1:10 in saline and then 20 µl thereof were mixed with 180 µl of 1/3 strength KB adjusted to pH 7.0 in 96-well plates (NUNC MicroWell™ 96-Well, Nunclon Delta-Treated, Flat-Bottom Microplate, Thermo Scientific, Waltham, MA, USA; Ref: 167008) resulting in a final OD_{600} of 0.005. Each treatment was added six times. The standard plate lids were replaced with peg-lids (NUNC Immuno TSP Lids, Thermo Scientific, Waltham, MA, USA; Ref: 445497). Plates were sealed with parafilm, and incubated at 24°C and 180 rpm. After 24 h, the biofilm formation was measured as described by Li et al. [9]. Briefly, biofilms grown on the pegs were washed by dipping pegs twice for 1 s in phosphate buffered saline (PBS; 8 g NaCl, 0.2 g KCl, 1.8 g Na_2HPO_4 x $2H_2O$, 0.245 g KH_2PO_4 dissolved in 1 l ddH_2O). Biofilms were stained for 20 min in 170 µl 0.1% crystal violet and then washed as described by dipping pegs twice for 1 s in PBS. Biofilms were incubated for 20 min in 200 µl 96% EtOH to dissolve the crystal violet and optical density as an approximation for biofilm formation was measured at 590 nm (OD_{590}) with a multimode microplate reader (Spark 10M, Tecan, Männedorf, Switzerland). To verify growth, OD_{600} of the 24 h old cultures was measured as well. Each replicate was measured three times. Each line was tested in two independent experiments.

In vitro growth assay

Media used for *in vitro* growth were 1/3 strength KB adjusted to pH 7.0 (KB pH 7.0), pH 5.5 (KB pH 5.5) for low pH stress or pH 8.5 (KB pH8.5) for high pH stress. For oxygen stress KB pH 7.0 was supplemented with 0.001% H_2O_2 (KB H_2O_2) shortly before use. Grace's insect medium (Sigma-Aldrich, St. Louis, MO, USA; Ref: 051M8307) was prepared according to the manufacturer's instructions and filter sterilised (0.22 µm, TPP, Trasadingen, Switzerland; Ref: 99722). *Pseudomonas* inoculums (OD_{600} = 0.5) were first diluted 1:10 in saline and then 20 µl thereof were mixed with 180 µl of the respective media in 96-well plates (NUNC MicroWell 96-Well, Nunclon Delta-Treated, Flat-Bottom Microplate, Thermo Scientific; Ref: 167008) resulting in a final OD_{600} of 0.005. Each treatment was added six times. Plates were sealed with parafilm and incubated at 24°C and 180 rpm. At 16, 24 and 48 hpi, OD_{600} as an approximation for bacterial growth was measured with a multimode microplate reader (Spark 10M, Tecan, Männedorf, Switzerland). Each replicate was measured three times. Each line was tested in three to four independent experiments.

Bibliography

[1] D Gaio, K Anantanawat, J To, M Liu, L Monahan, and AE Darling, 2022. Hackflex: Low-cost, high-throughput, Illumina Nextera Flex library construction. *Microb. Genom.*, 8, doi:10.1099/MGEN.0.000744.

[2] J Huerta-Cepas, D Szklarczyk, D Heller, A Hernández-Plaza, SK Forslund, H Cook, DR Mende, I Letunic, T Rattei, LJ Jensen, C Von Mering, and P Bork, 2019. eggNOG 5.0: a hierarchical, functionally and phylogenetically annotated orthology resource based on 5090 organisms and 2502 viruses. *Nucleic Acids Res.*, 47, doi:10.1093/NAR/GKY1085.

[3] DE Wood, J Lu, and B Langmead, 2019. Improved metagenomic analysis with Kraken 2. *Genome Biol.*, 20, doi:10.1186/s13059-019-1891-0.

[4] EO King, MK Ward, and DE Raney, 1954. Two simple media for the demonstration of pyocyanin and fluorescin. *J. Lab. Clin. Med.*, 44, doi:10.5555/URI:PII:002221435490222X.

[5] P Vesga, E Augustiny, C Keel, M Maurhofer, and J Vacheron, 2021. Phylogenetically closely related pseudomonads isolated from arthropods exhibit differential insect-killing abilities and genetic variations in insecticidal factors. *Environ. Microbiol.*, 23, doi:10.1111/1462-2920.15623.

[6] P Flury, P Vesga, M Péchy-Tarr, N Aellen, F Dennert, N Hofer, KP Kupferschmied, P Kupferschmied, Z Metla, Z Ma, S Siegfried, S de Weert, G Bloemberg, M Höfte, CJ Keel, and M Maurhofer, 2017. Antimicrobial and insecticidal: Cyclic lipopeptides and hydrogen cyanide produced by plant-beneficial Pseudomonas strains CHA0, CMR12a, and PCL1391 contribute to insect killing. *Front. Microbiol.*, 8, doi:10.3389/fmicb.2017.00100.

[7] DJ Lane, 1991. 16S/23S rRNA sequencing. In E Stackebrandt and M Goodfellow, editors, *Nucleic acid techniques in bacterial systematics*, pages 115–175. John Wiley and Sons, New York.

[8] JJ Sánchez-Gil, SW Poppeliers, J Vacheron, H Zhang, B Odijk, C Keel, and R de Jonge, 2023. The conserved iol gene cluster in Pseudomonas is involved in rhizosphere competence. *Curr. Biol.*, 33, doi:10.1016/J.CUB.2023.05.057.

[9] E Li, R de Jonge, C Liu, H Jiang, VP Friman, CM Pieterse, PA Bakker, and A Jousset, 2021. Rapid evolution of bacterial mutualism in the plant rhizosphere. *Nat. Commun.*, 12, doi:10.1038/s41467-021-24005-y.
