# Supplementary material for: The insect- and plant-associated lifestyles of Pseudomonas protegens CHA0 are preserved following serial passage through insect larvae
Source: PLoS Pathog. 2026 Sep 21;22(9):e1014101. doi: 10.1371/journal.ppat.1014101 (PMC13592716; doi:10.1371/journal.ppat.1014101)
Supplement: Supplementary file 3 [file ppat.1014101.s006.pdf]

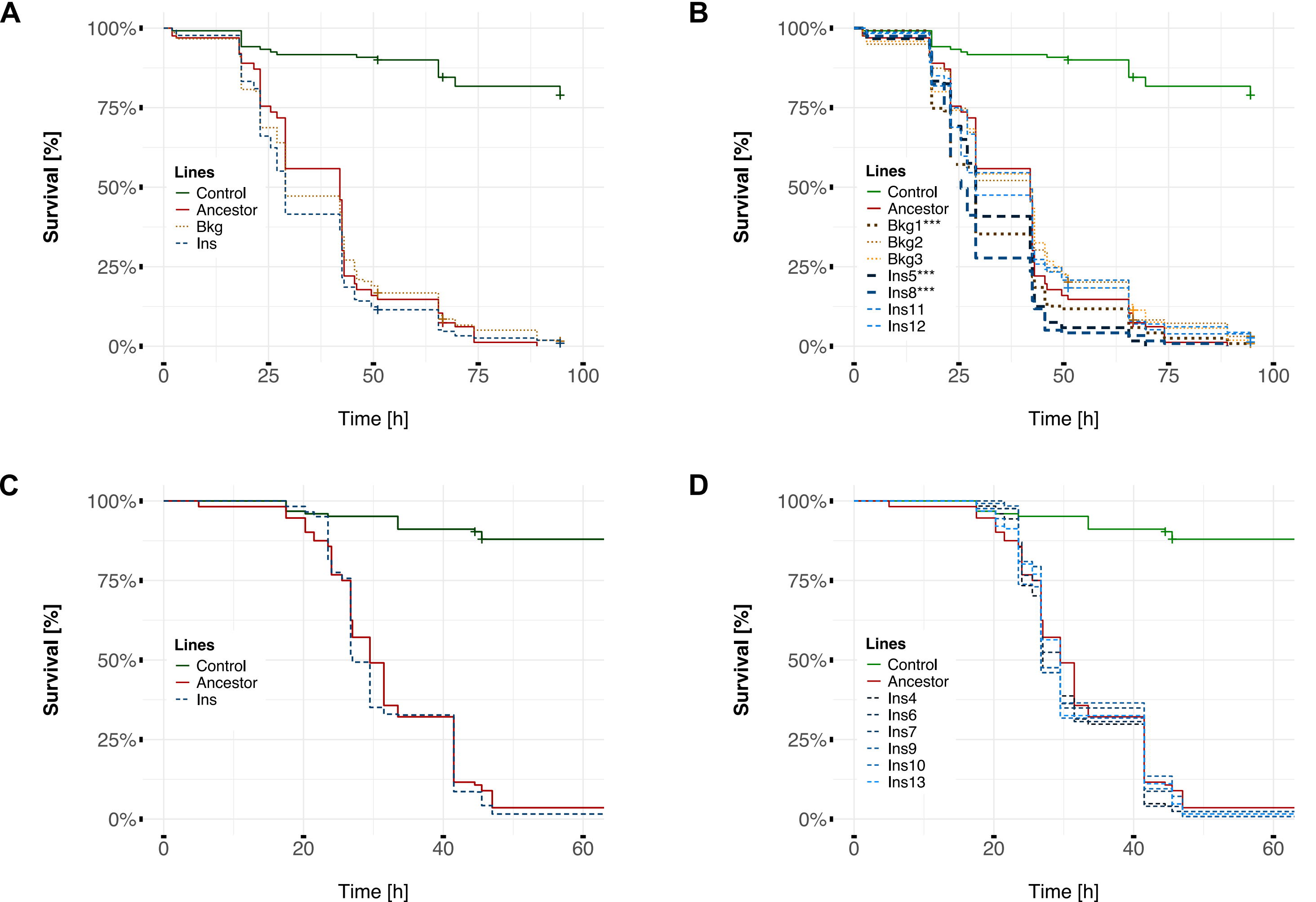

A
B
C
D
Survival [%]
100%
75%
50%
25%
0%
Time [h]
0
25
50
75
100
20
40
60
Lines
Control
Ancestor
Bkg
Ins
Bkg1***
Bkg2
Bkg3
Ins5***
Ins8***
Ins11
Ins12
Ins4
Ins6
Ins7
Ins9
Ins10
Ins13
