# Supplementary material for: The insect- and plant-associated lifestyles of Pseudomonas protegens CHA0 are preserved following serial passage through insect larvae
Source: PLoS Pathog. 2026 Sep 21;22(9):e1014101. doi: 10.1371/journal.ppat.1014101 (PMC13592716; doi:10.1371/journal.ppat.1014101)
Supplement: Supplementary file 4 [file ppat.1014101.s007.pdf]

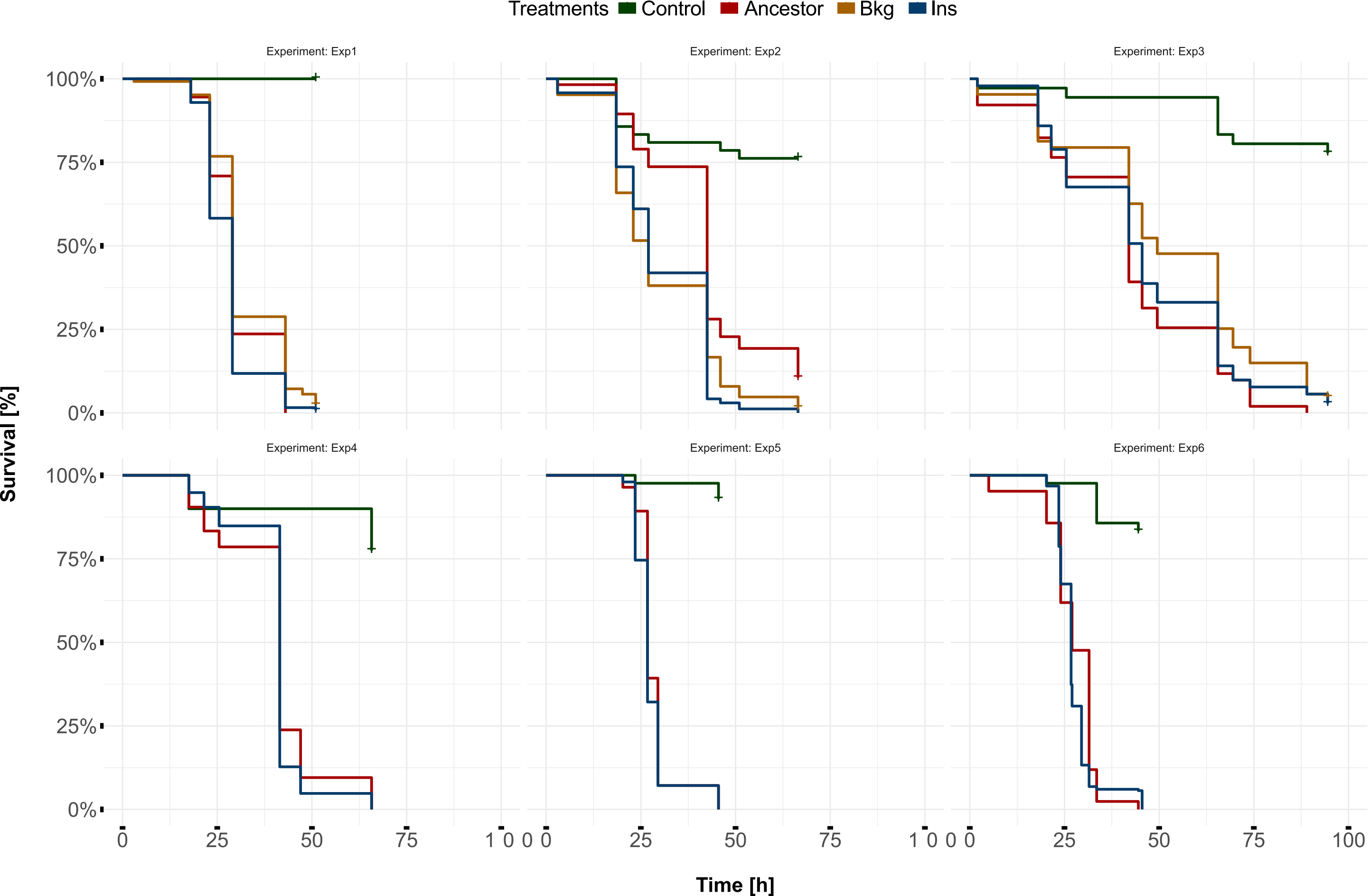

Treatments
Control
Ancestor
Bkg
Ins
Experiment: Exp1
Experiment: Exp2
Experiment: Exp3
Experiment: Exp4
Experiment: Exp5
Experiment: Exp6
100%
75%
50%
25%
0%
0
25
50
75
100
Survival [%]
Time [h]
