# Supplementary material for: The insect- and plant-associated lifestyles of Pseudomonas protegens CHA0 are preserved following serial passage through insect larvae
Source: PLoS Pathog. 2026 Sep 21;22(9):e1014101. doi: 10.1371/journal.ppat.1014101 (PMC13592716; doi:10.1371/journal.ppat.1014101)
Supplement: Supplementary file 5 [file ppat.1014101.s008.pdf]

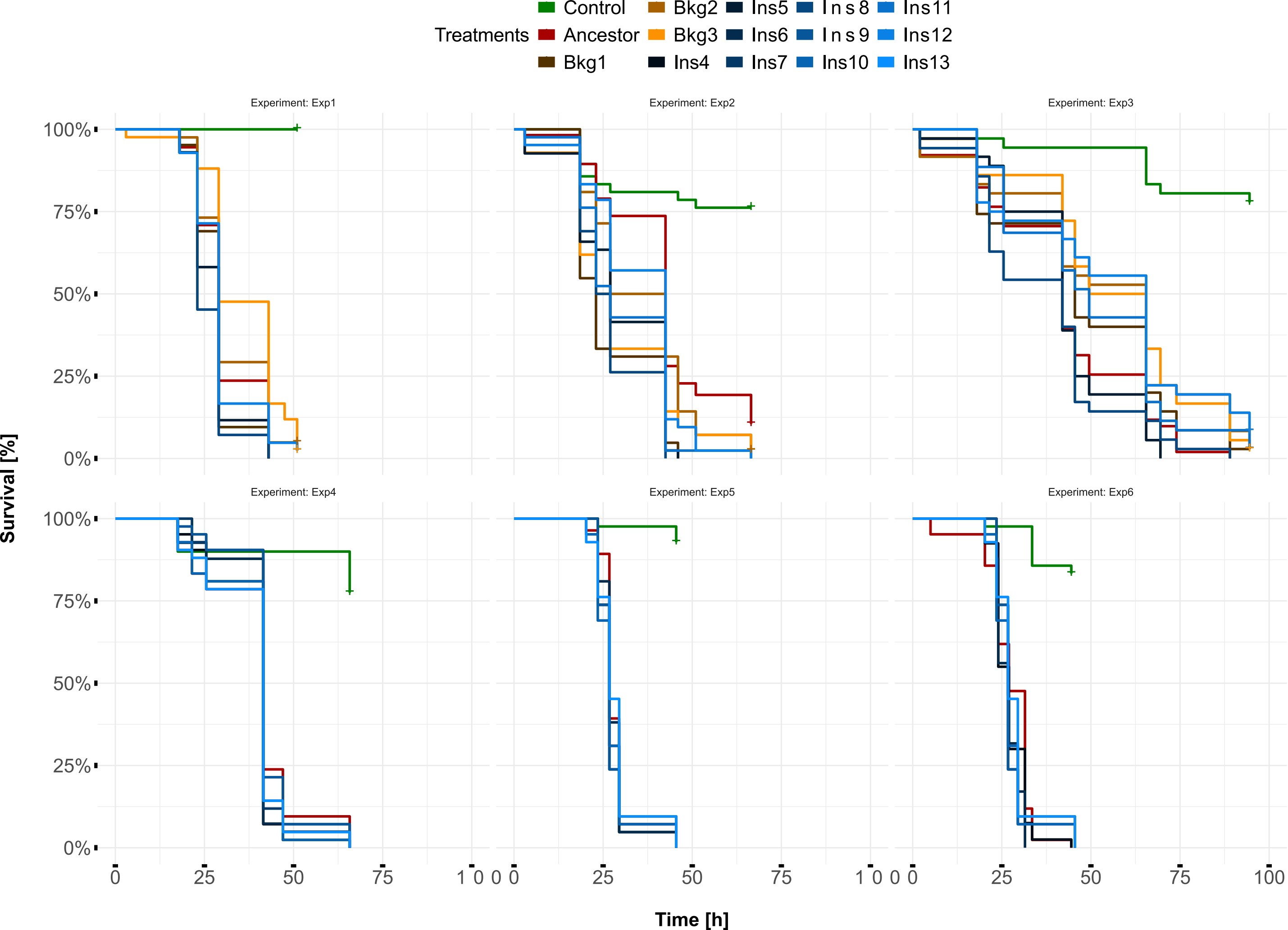
Treatments
Control
Ancestor
Bkg1
Bkg2
Bkg3
Ins4
Ins5
Ins6
Ins7
Ins8
Ins9
Ins10
Ins11
Ins12
Ins13
Experiment: Exp1
Experiment: Exp2
Experiment: Exp3
Experiment: Exp4
Experiment: Exp5
Experiment: Exp6
100%
75%
50%
25%
0%
Survival [%]
0
25
50
75
100
Time [h]
