# Supplementary material for: The insect- and plant-associated lifestyles of Pseudomonas protegens CHA0 are preserved following serial passage through insect larvae
Source: PLoS Pathog. 2026 Sep 21;22(9):e1014101. doi: 10.1371/journal.ppat.1014101 (PMC13592716; doi:10.1371/journal.ppat.1014101)
Supplement: Supplementary file 6 [file ppat.1014101.s009.pdf]

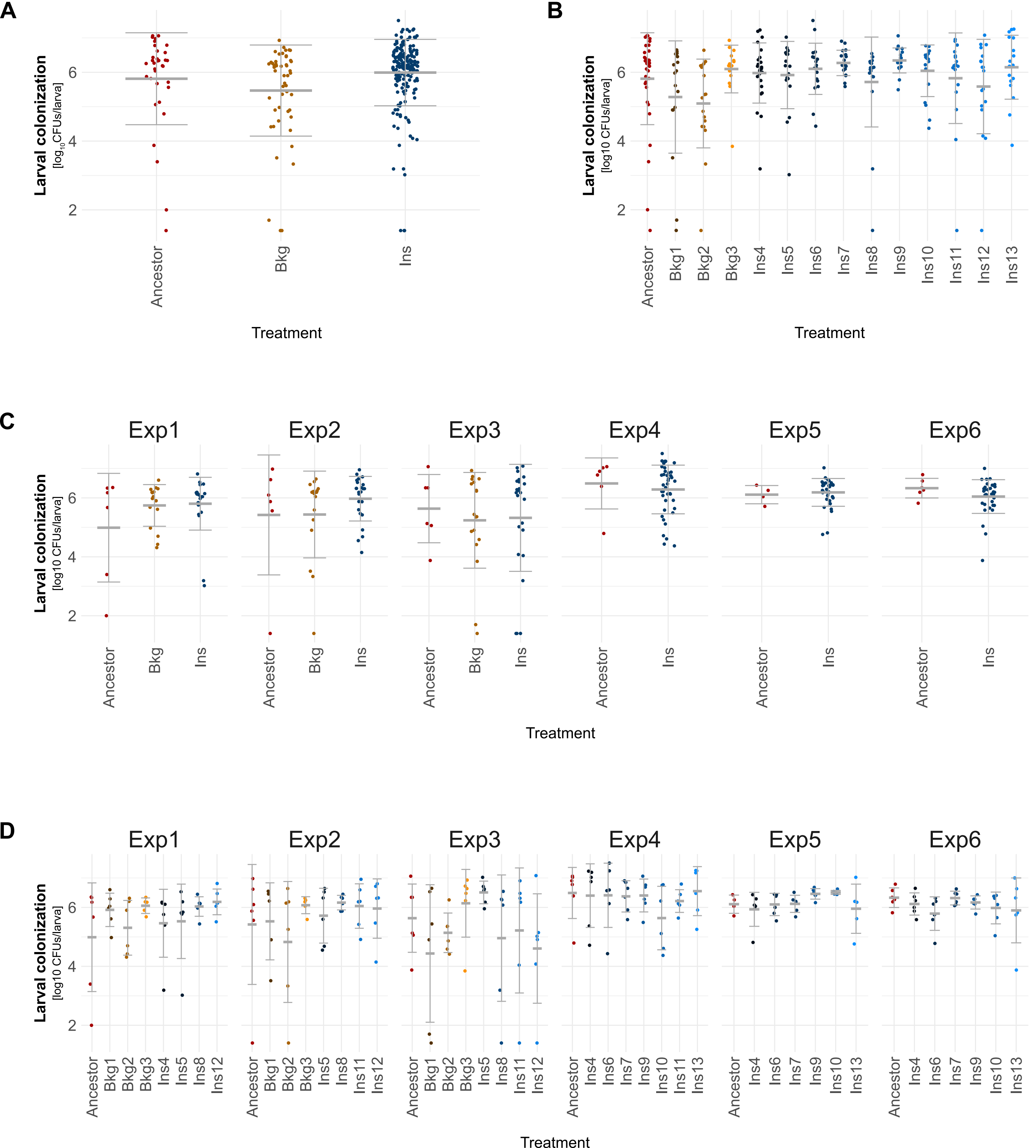

A
Larval colonization
[log10CFUs/larva]
6
4
2
Ancestor
Bkg
Ins
Treatment
B
Larval colonization
[log10 CFUs/larva]
Ancestor
Bkg1
Bkg2
Bkg3
Ins4
Ins5
Ins6
Ins7
Ins8
Ins9
Ins10
Ins11
Ins12
Ins13
Treatment
C
Exp1
Exp2
Exp3
Exp4
Exp5
Exp6
Larval colonization
[log10 CFUs/larva]
Ancestor
Bkg
Ins
Treatment
D
Exp1
Exp2
Exp3
Exp4
Exp5
Exp6
Larval colonization
[log10 CFUs/larva]
Ancestor
Bkg1
Bkg2
Bkg3
Ins4
Ins5
Ins8
Ins12
Ins11
Ins6
Ins7
Ins9
Ins10
Ins13
Treatment
