# Supplementary material for: The insect- and plant-associated lifestyles of Pseudomonas protegens CHA0 are preserved following serial passage through insect larvae
Source: PLoS Pathog. 2026 Sep 21;22(9):e1014101. doi: 10.1371/journal.ppat.1014101 (PMC13592716; doi:10.1371/journal.ppat.1014101)
Supplement: Supplementary file 7 [file ppat.1014101.s010.pdf]

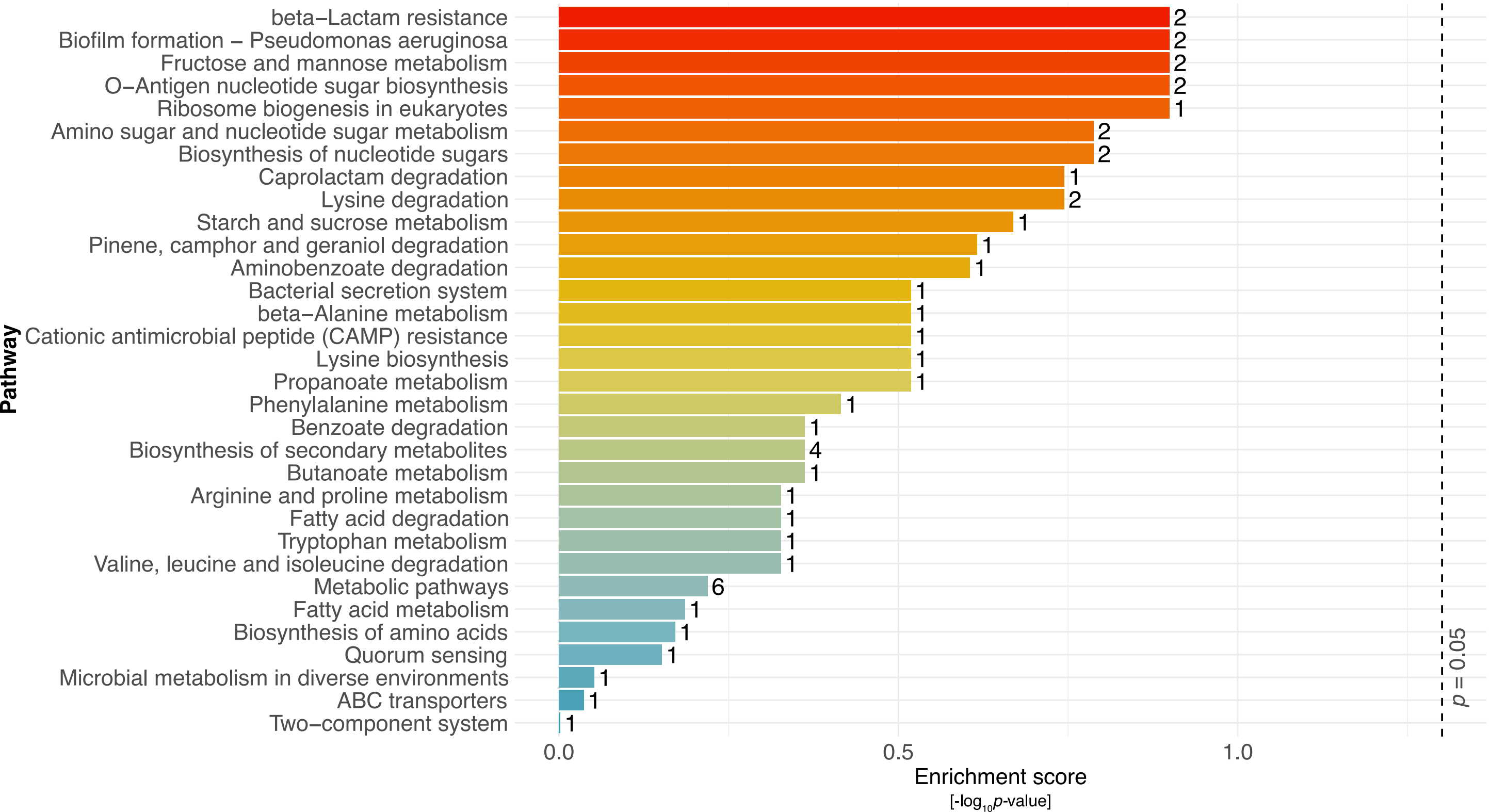
Pathway
beta−Lactam resistance 2
Biofilm formation − Pseudomonas aeruginosa 2
Fructose and mannose metabolism 2
O−Antigen nucleotide sugar biosynthesis 2
Ribosome biogenesis in eukaryotes 1
Amino sugar and nucleotide sugar metabolism 2
Biosynthesis of nucleotide sugars 2
Caprolactam degradation 1
Lysine degradation 2
Starch and sucrose metabolism 1
Pinene, camphor and geraniol degradation 1
Aminobenzoate degradation 1
Bacterial secretion system 1
beta−Alanine metabolism 1
Cationic antimicrobial peptide (CAMP) resistance 1
Lysine biosynthesis 1
Propanoate metabolism 1
Phenylalanine metabolism 1
Benzoate degradation 1
Biosynthesis of secondary metabolites 4
Butanoate metabolism 1
Arginine and proline metabolism 1
Fatty acid degradation 1
Tryptophan metabolism 1
Valine, leucine and isoleucine degradation 1
Metabolic pathways 6
Fatty acid metabolism 1
Biosynthesis of amino acids 1
Quorum sensing 1
Microbial metabolism in diverse environments 1
ABC transporters 1
Two−component system 1
p = 0.05
0.0
0.5
1.0
Enrichment score
[-log10 p-value]
