# Supplementary material for: The insect- and plant-associated lifestyles of Pseudomonas protegens CHA0 are preserved following serial passage through insect larvae
Source: PLoS Pathog. 2026 Sep 21;22(9):e1014101. doi: 10.1371/journal.ppat.1014101 (PMC13592716; doi:10.1371/journal.ppat.1014101)
Supplement: Supplementary file 11 [file ppat.1014101.s014.pdf]

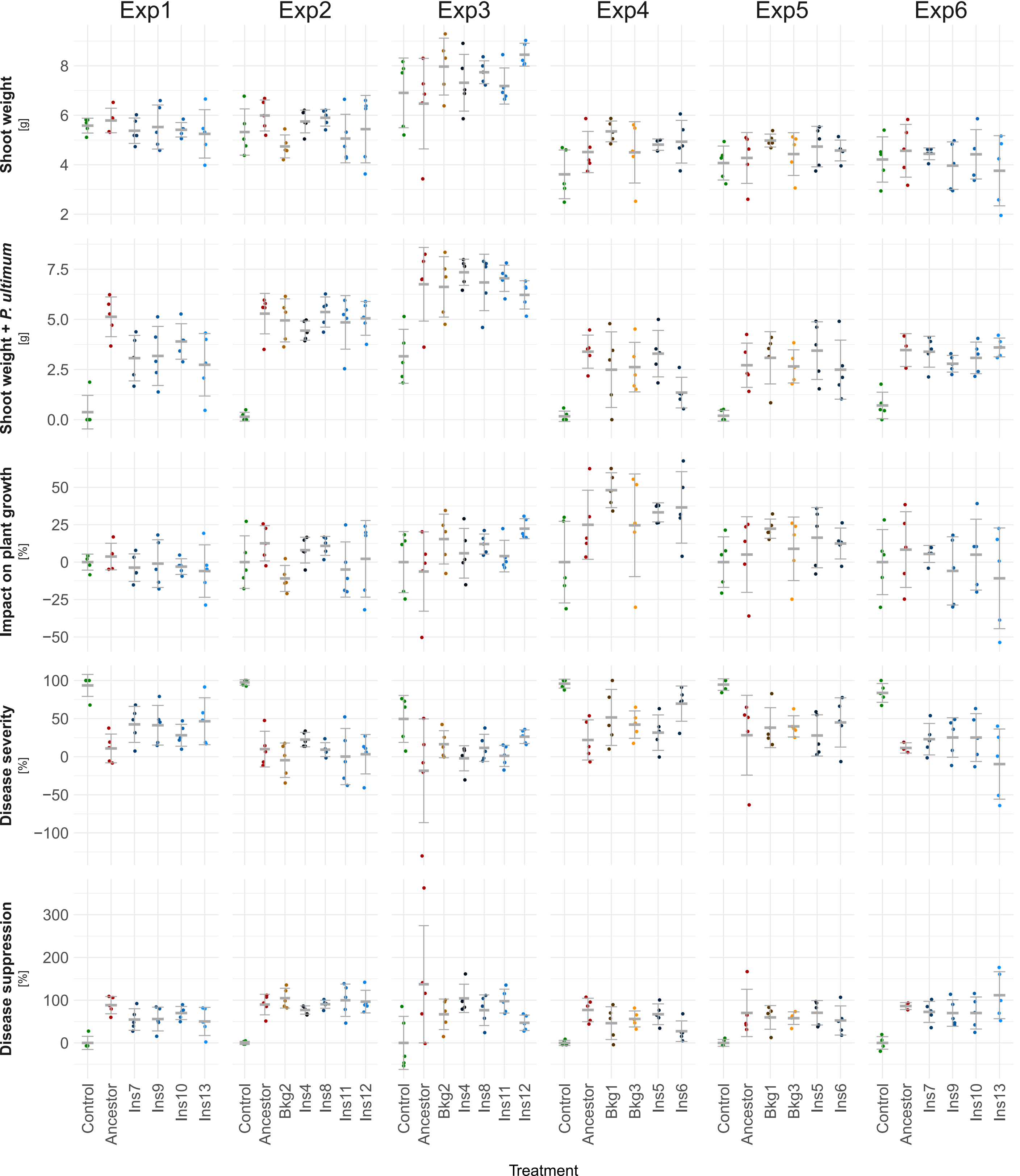

Exp1
Exp2
Exp3
Exp4
Exp5
Exp6
Shoot weight [g]
Shoot weight + P. ultimum [g]
Impact on plant growth [%]
Disease severity [%]
Disease suppression [%]
Control
Ancestor
Ins7
Ins9
Ins10
Ins13
Bkg2
Ins4
Ins8
Ins11
Ins12
Bkg1
Bkg3
Ins5
Ins6
Treatment
