# Supplementary material for: The insect- and plant-associated lifestyles of Pseudomonas protegens CHA0 are preserved following serial passage through insect larvae
Source: PLoS Pathog. 2026 Sep 21;22(9):e1014101. doi: 10.1371/journal.ppat.1014101 (PMC13592716; doi:10.1371/journal.ppat.1014101)
Supplement: Supplementary file 12 [file ppat.1014101.s015.pdf]

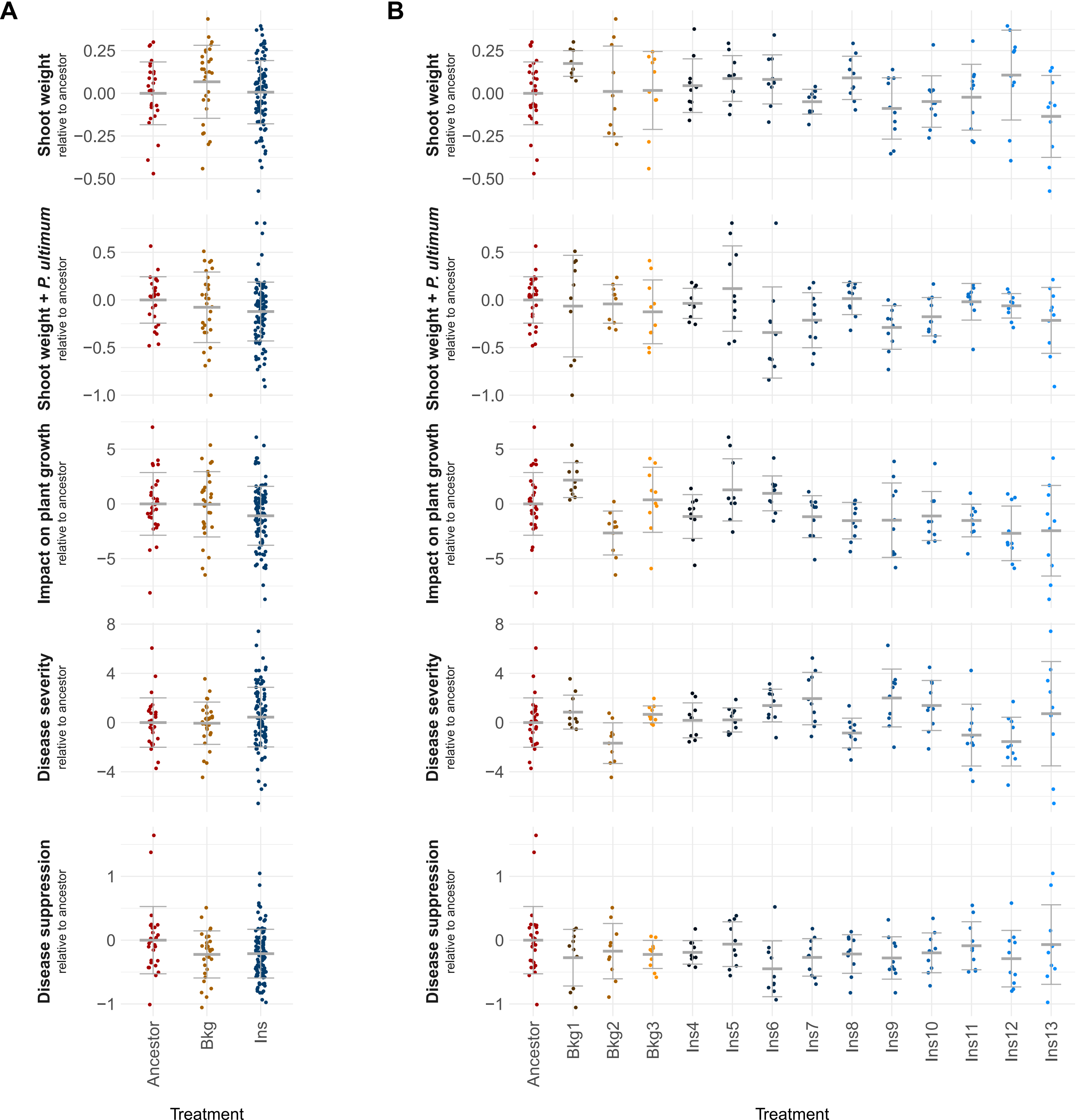

A
B
Shoot weight
relative to ancestor
Shoot weight + P. ultimum
relative to ancestor
Impact on plant growth
relative to ancestor
Disease severity
relative to ancestor
Disease suppression
relative to ancestor
0.25
0.00
−0.25
−0.50
0.5
0.0
−0.5
−1.0
5
0
−5
8
4
0
−4
1
0
−1
Ancestor
Bkg
Ins
Bkg1
Bkg2
Bkg3
Ins4
Ins5
Ins6
Ins7
Ins8
Ins9
Ins10
Ins11
Ins12
Ins13
Treatment
