# Supplementary material for: The insect- and plant-associated lifestyles of Pseudomonas protegens CHA0 are preserved following serial passage through insect larvae
Source: PLoS Pathog. 2026 Sep 21;22(9):e1014101. doi: 10.1371/journal.ppat.1014101 (PMC13592716; doi:10.1371/journal.ppat.1014101)
Supplement: Supplementary file 15 [file ppat.1014101.s018.pdf]

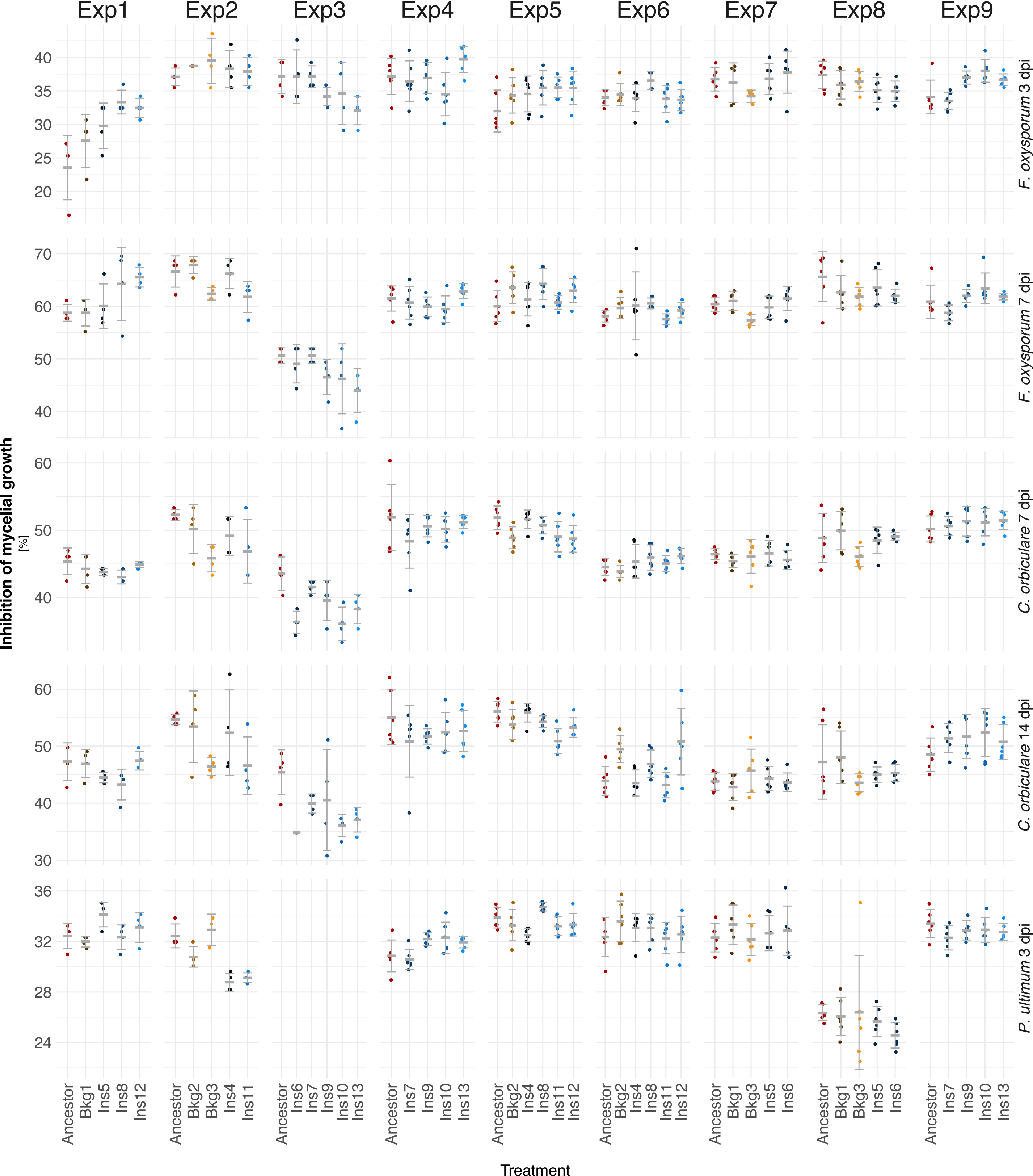

Exp1
Exp2
Exp3
Exp4
Exp5
Exp6
Exp7
Exp8
Exp9
F. oxysporum 3 dpi
F. oxysporum 7 dpi
C. orbiculare 7 dpi
C. orbiculare 14 dpi
P. ultimum 3 dpi
Inhibition of mycelial growth
[%]
Treatment
Ancestor
Bkg1
Bkg2
Bkg3
Ins4
Ins5
Ins6
Ins7
Ins8
Ins9
Ins10
Ins11
Ins12
Ins13
