# Supplementary material for: The insect- and plant-associated lifestyles of Pseudomonas protegens CHA0 are preserved following serial passage through insect larvae
Source: PLoS Pathog. 2026 Sep 21;22(9):e1014101. doi: 10.1371/journal.ppat.1014101 (PMC13592716; doi:10.1371/journal.ppat.1014101)
Supplement: Supplementary file 21 [file ppat.1014101.s024.pdf]

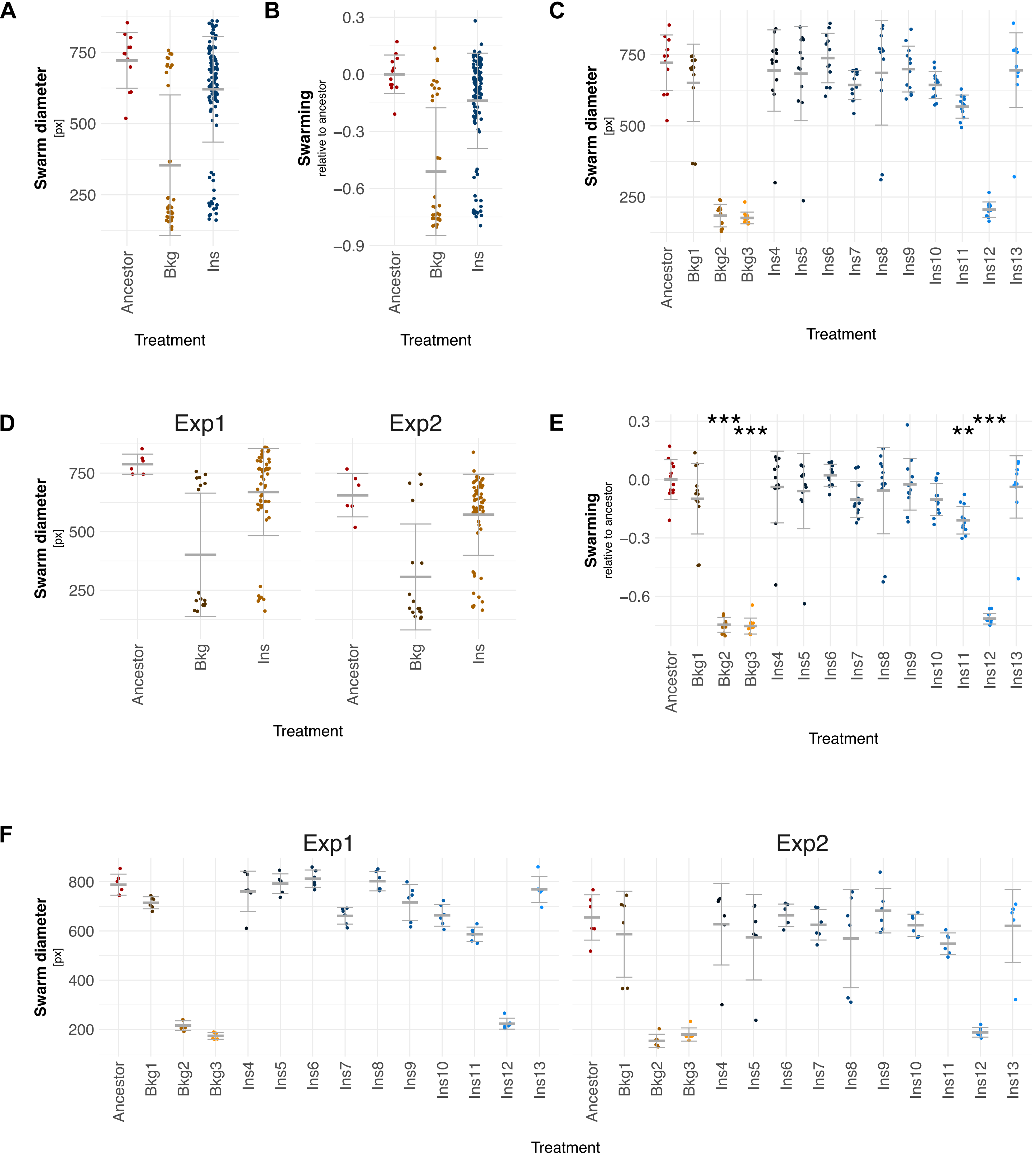

A
Swarm diameter [px]
750
500
250
Ancestor
Bkg
Ins
Treatment
B
Swarming relative to ancestor
0.3
0.0
−0.3
−0.6
−0.9
Ancestor
Bkg
Ins
Treatment
C
Swarm diameter [px]
750
500
250
Ancestor
Bkg1
Bkg2
Bkg3
Ins4
Ins5
Ins6
Ins7
Ins8
Ins9
Ins10
Ins11
Ins12
Ins13
Treatment
D
Exp1
Exp2
Swarm diameter [px]
750
500
250
Ancestor
Bkg
Ins
Ancestor
Bkg
Ins
Treatment
E
Swarming relative to ancestor
0.3
0.0
−0.3
−0.6
***
***
**
***
Ancestor
Bkg1
Bkg2
Bkg3
Ins4
Ins5
Ins6
Ins7
Ins8
Ins9
Ins10
Ins11
Ins12
Ins13
Treatment
F
Exp1
Exp2
Swarm diameter [px]
800
600
400
200
Ancestor
Bkg1
Bkg2
Bkg3
Ins4
Ins5
Ins6
Ins7
Ins8
Ins9
Ins10
Ins11
Ins12
Ins13
Ancestor
Bkg1
Bkg2
Bkg3
Ins4
Ins5
Ins6
Ins7
Ins8
Ins9
Ins10
Ins11
Ins12
Ins13
Treatment
