# Supplementary material for: The insect- and plant-associated lifestyles of Pseudomonas protegens CHA0 are preserved following serial passage through insect larvae
Source: PLoS Pathog. 2026 Sep 21;22(9):e1014101. doi: 10.1371/journal.ppat.1014101 (PMC13592716; doi:10.1371/journal.ppat.1014101)
Supplement: Supplementary file 25 [file ppat.1014101.s028.pdf]

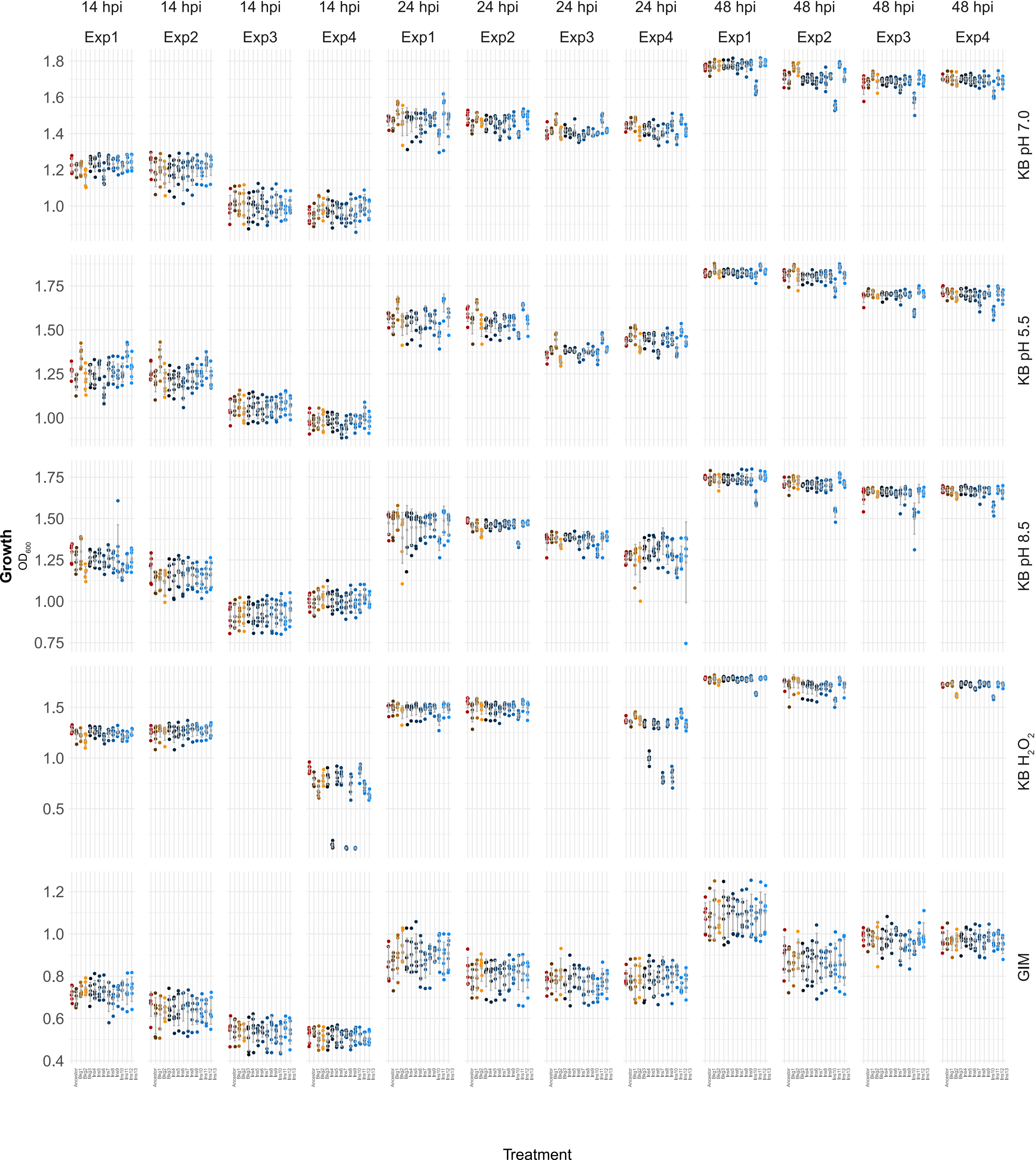
14 hpi
24 hpi
48 hpi
Exp1
Exp2
Exp3
Exp4
KB pH 7.0
KB pH 5.5
KB pH 8.5
KB H_2O_2
GIM
Growth
OD_{600}
Ancestor
Bkg1
Bkg2
Bkg3
Ins4
Ins5
Ins6
Ins7
Ins8
Ins9
Ins10
Ins11
Ins12
Ins13
Treatment
