# Supplementary material for: The insect- and plant-associated lifestyles of Pseudomonas protegens CHA0 are preserved following serial passage through insect larvae
Source: PLoS Pathog. 2026 Sep 21;22(9):e1014101. doi: 10.1371/journal.ppat.1014101 (PMC13592716; doi:10.1371/journal.ppat.1014101)
Supplement: Supplementary file 26 [file ppat.1014101.s029.pdf]

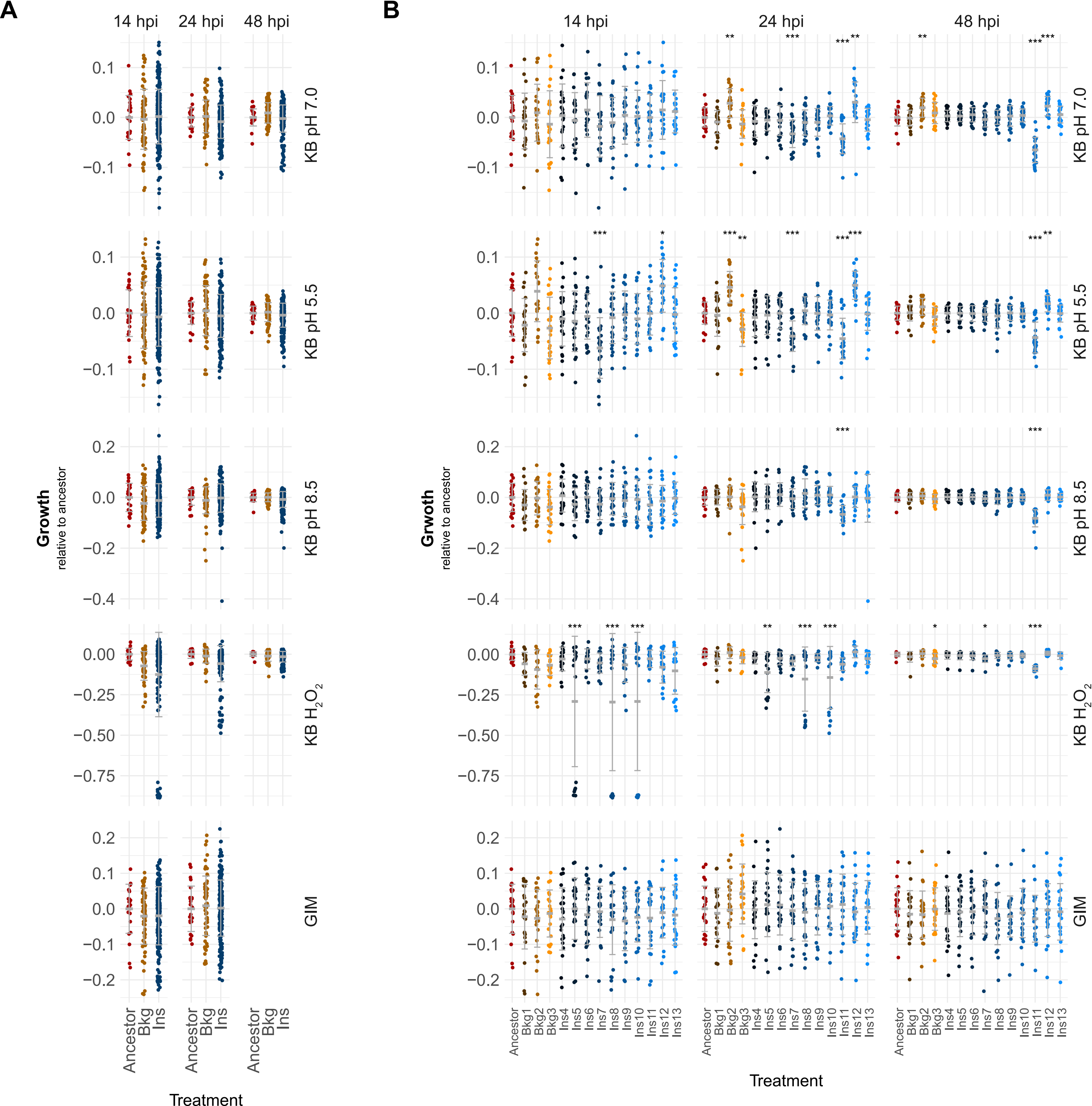

A
B
14 hpi
24 hpi
48 hpi
KB pH 7.0
KB pH 5.5
KB pH 8.5
KB H2O2
GIM
Growth
relative to ancestor
Grwoth
Treatment
Ancestor
Bkg
Ins
Bkg1
Bkg2
Bkg3
Ins4
Ins5
Ins6
Ins7
Ins8
Ins9
Ins10
Ins11
Ins12
Ins13
