# Supplementary material for: The insect- and plant-associated lifestyles of Pseudomonas protegens CHA0 are preserved following serial passage through insect larvae
Source: PLoS Pathog. 2026 Sep 21;22(9):e1014101. doi: 10.1371/journal.ppat.1014101 (PMC13592716; doi:10.1371/journal.ppat.1014101)
Supplement: Supplementary file 27 [file ppat.1014101.s030.pdf]

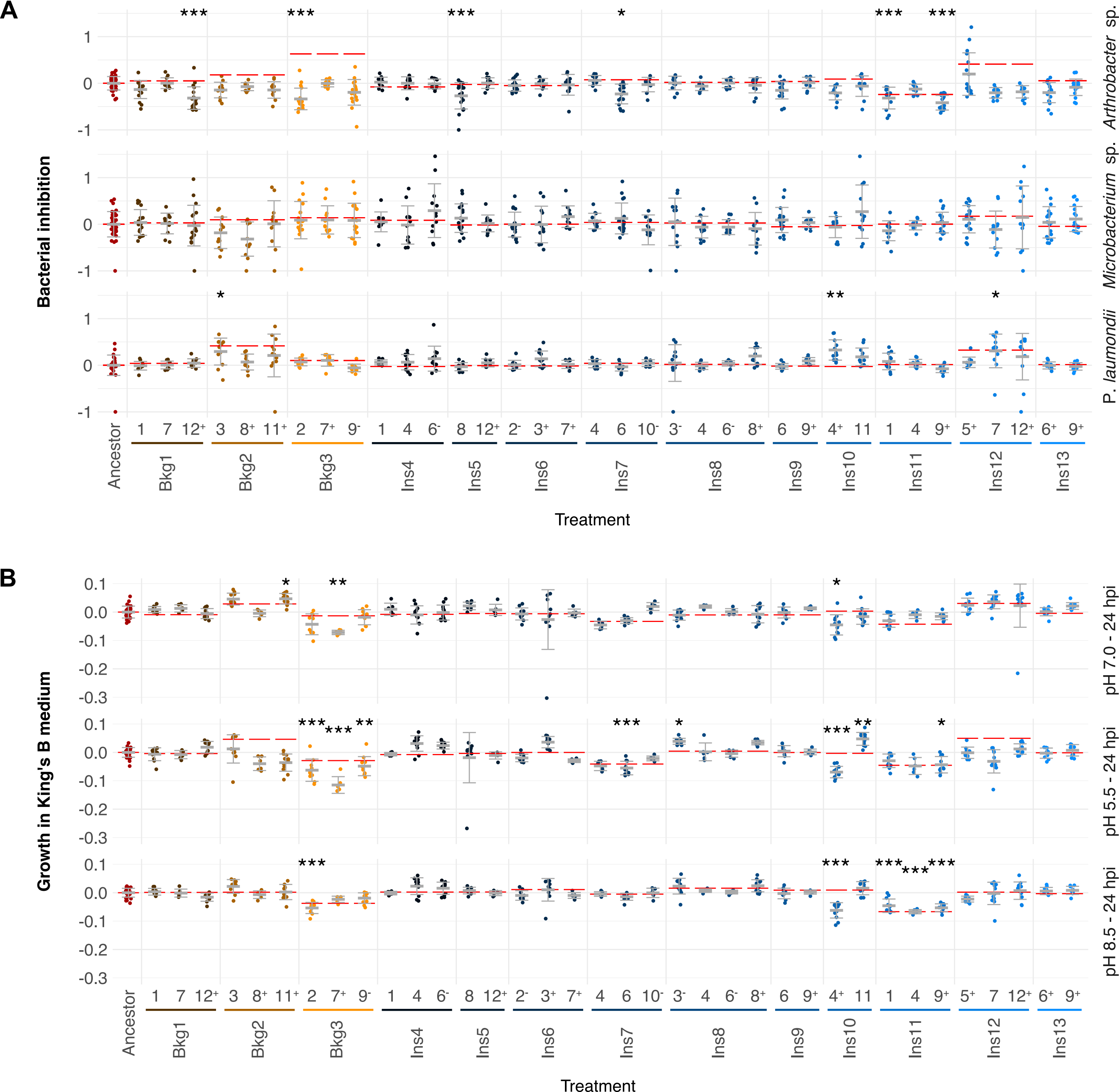

A
Bacterial inhibition
Arthrobacter sp.
Microbacterium sp.
P. laumondii
Treatment
Ancestor
Bkg1
Bkg2
Bkg3
Ins4
Ins5
Ins6
Ins7
Ins8
Ins9
Ins10
Ins11
Ins12
Ins13
B
Growth in King's B medium
pH 7.0 - 24 hpi
pH 5.5 - 24 hpi
pH 8.5 - 24 hpi
Treatment
