# Supplementary material for: The insect- and plant-associated lifestyles of Pseudomonas protegens CHA0 are preserved following serial passage through insect larvae
Source: PLoS Pathog. 2026 Sep 21;22(9):e1014101. doi: 10.1371/journal.ppat.1014101 (PMC13592716; doi:10.1371/journal.ppat.1014101)
Supplement: Supplementary file 28 [file ppat.1014101.s031.pdf]

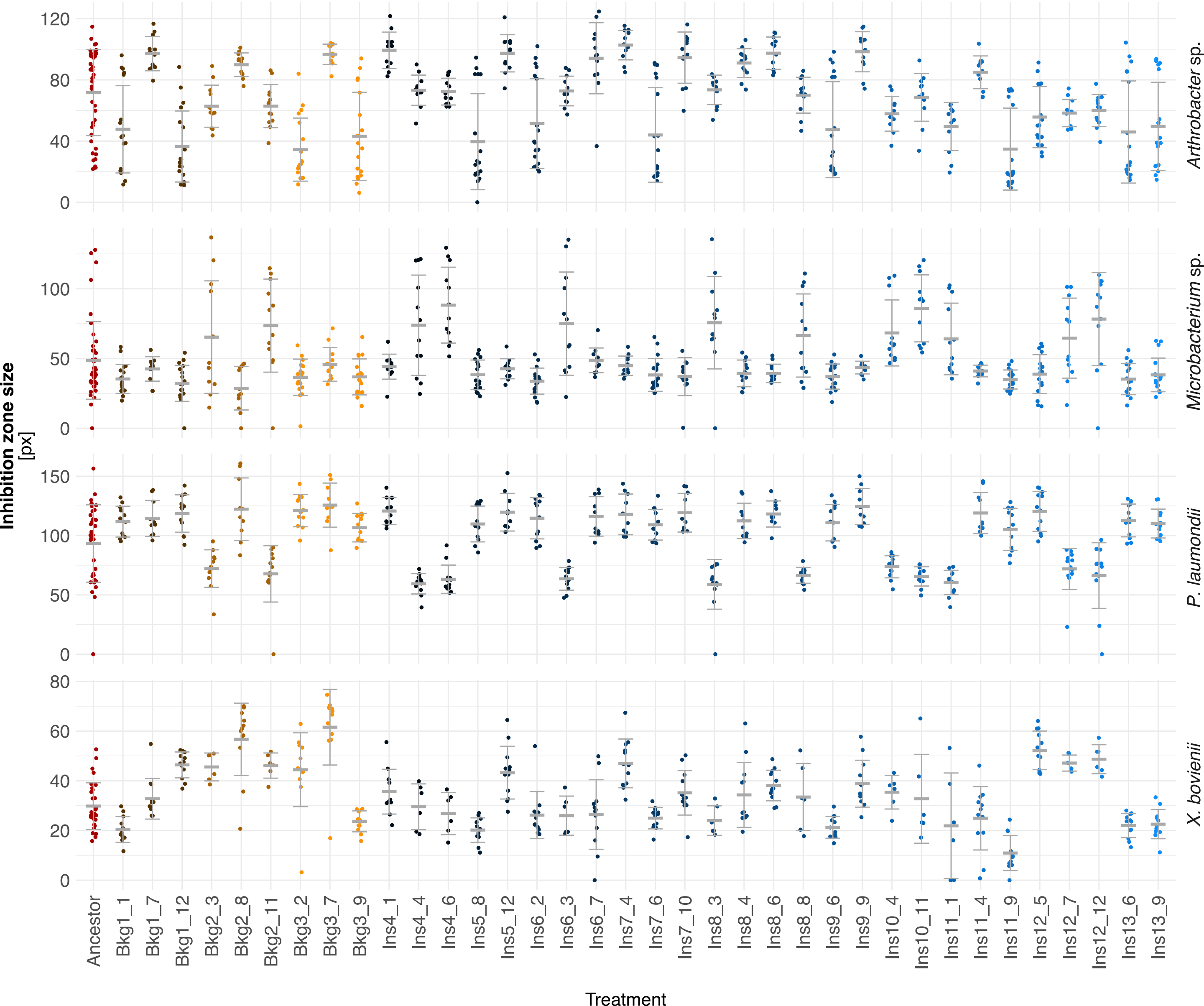

Inhibition zone size
[px]
Treatment
Arthrobacter sp.
Microbacterium sp.
P. laumondii
X. bovienii
Ancestor
Bkg1_1
Bkg1_7
Bkg1_12
Bkg2_3
Bkg2_8
Bkg2_11
Bkg3_2
Bkg3_7
Bkg3_9
Ins4_1
Ins4_4
Ins4_6
Ins5_8
Ins5_12
Ins6_2
Ins6_3
Ins6_7
Ins7_4
Ins7_6
Ins7_10
Ins8_3
Ins8_4
Ins8_6
Ins8_8
Ins9_6
Ins9_9
Ins10_4
Ins10_11
Ins11_1
Ins11_4
Ins11_9
Ins12_5
Ins12_7
Ins12_12
Ins13_6
Ins13_9
