# Supplementary material for: The insect- and plant-associated lifestyles of Pseudomonas protegens CHA0 are preserved following serial passage through insect larvae
Source: PLoS Pathog. 2026 Sep 21;22(9):e1014101. doi: 10.1371/journal.ppat.1014101 (PMC13592716; doi:10.1371/journal.ppat.1014101)
Supplement: Supplementary file 29 [file ppat.1014101.s032.pdf]

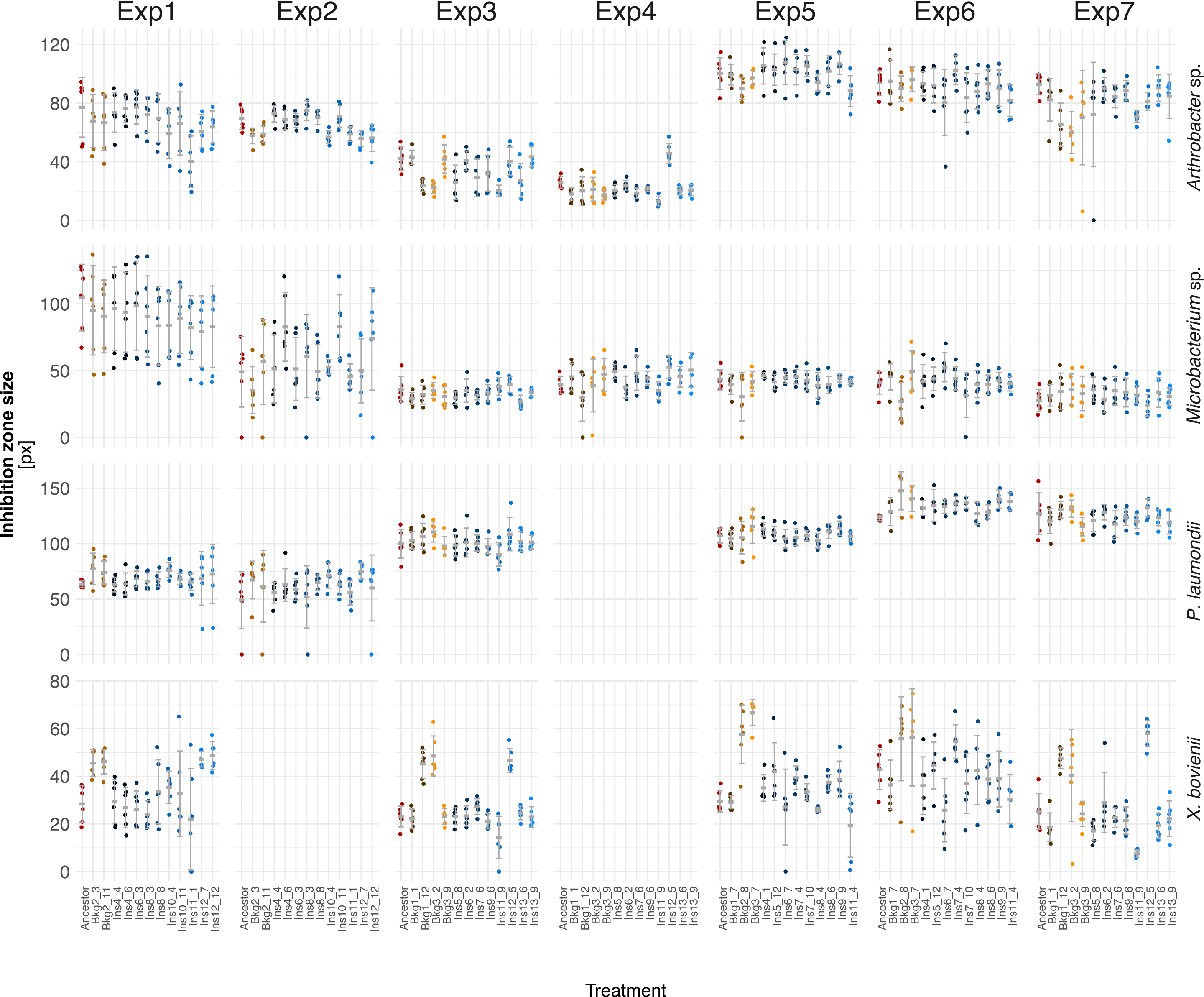

Exp1
Exp2
Exp3
Exp4
Exp5
Exp6
Exp7
Arthrobacter sp.
Microbacterium sp.
P. laumondii
X. bovienii
Inhibition zone size
[px]
Treatment
