# Supplementary material for: The insect- and plant-associated lifestyles of Pseudomonas protegens CHA0 are preserved following serial passage through insect larvae
Source: PLoS Pathog. 2026 Sep 21;22(9):e1014101. doi: 10.1371/journal.ppat.1014101 (PMC13592716; doi:10.1371/journal.ppat.1014101)
Supplement: Supplementary file 33 [file ppat.1014101.s036.pdf]

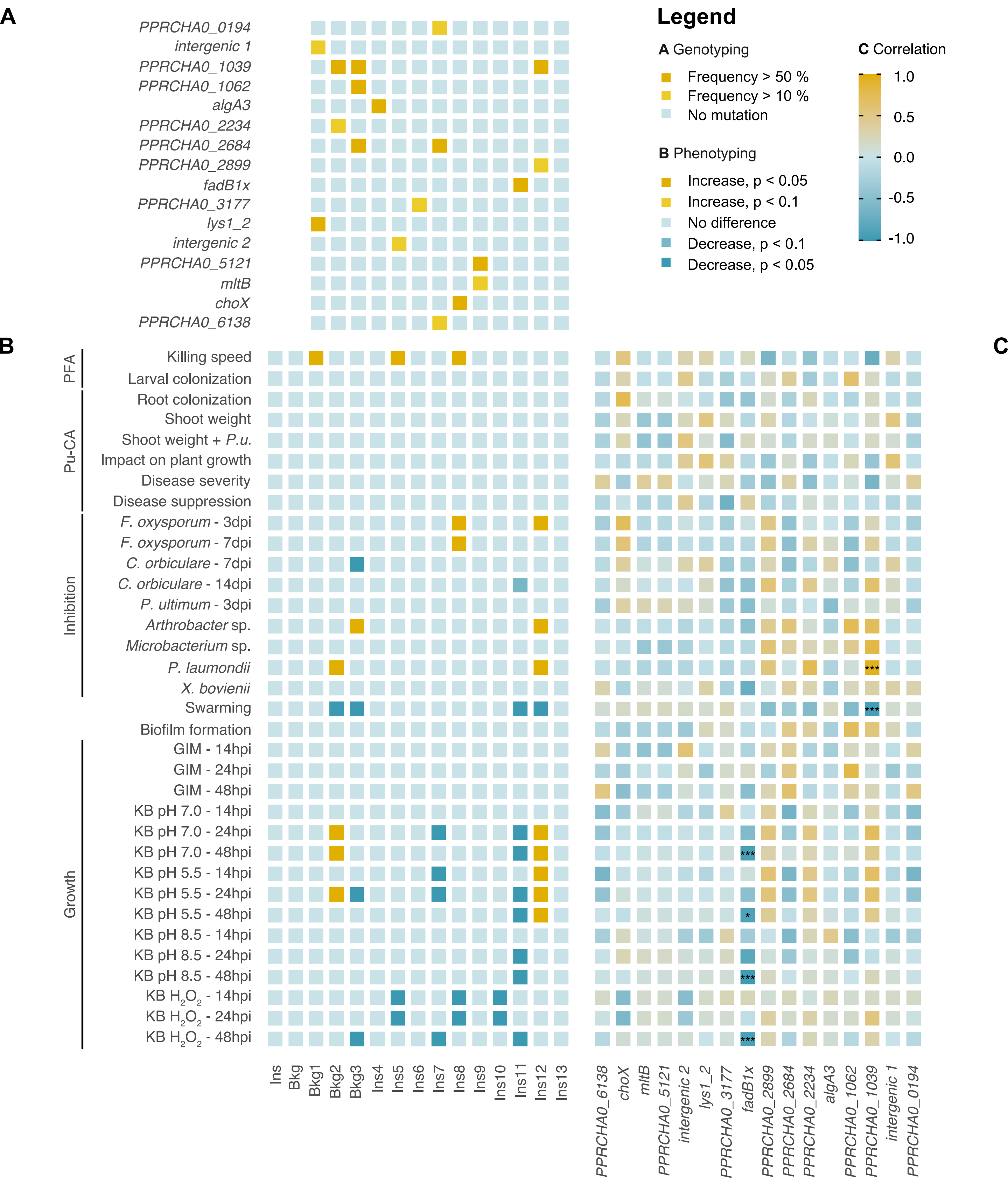
A
B
C
Legend
A Genotyping
Frequency > 50 %
Frequency > 10 %
No mutation
B Phenotyping
Increase, p < 0.05
Increase, p < 0.1
No difference
Decrease, p < 0.1
Decrease, p < 0.05
C Correlation
1.0
0.5
0.0
-0.5
-1.0
PPRCHA0_0194
intergenic 1
PPRCHA0_1039
PPRCHA0_1062
algA3
PPRCHA0_2234
PPRCHA0_2684
PPRCHA0_2899
fadB1x
PPRCHA0_3177
lys1_2
intergenic 2
PPRCHA0_5121
mltB
choX
PPRCHA0_6138
PFA
Pu-CA
Inhibition
Growth
Killing speed
Larval colonization
Root colonization
Shoot weight
Shoot weight + P.u.
Impact on plant growth
Disease severity
Disease suppression
F. oxysporum - 3dpi
F. oxysporum - 7dpi
C. orbiculare - 7dpi
C. orbiculare - 14dpi
P. ultimum - 3dpi
Arthrobacter sp.
Microbacterium sp.
P. laumondii
X. bovienii
Swarming
Biofilm formation
GIM - 14hpi
GIM - 24hpi
GIM - 48hpi
KB pH 7.0 - 14hpi
KB pH 7.0 - 24hpi
KB pH 7.0 - 48hpi
KB pH 5.5 - 14hpi
KB pH 5.5 - 24hpi
KB pH 5.5 - 48hpi
KB pH 8.5 - 14hpi
KB pH 8.5 - 24hpi
KB pH 8.5 - 48hpi
KB H2O2 - 14hpi
KB H2O2 - 24hpi
KB H2O2 - 48hpi
***
*
Ins
Bkg
Bkg1
Bkg2
Bkg3
Ins4
Ins5
Ins6
Ins7
Ins8
Ins9
Ins10
Ins11
Ins12
Ins13
PPRCHA0_6138
choX
mltB
PPRCHA0_5121
intergenic 2
lys1_2
PPRCHA0_3177
fadB1x
PPRCHA0_2899
PPRCHA0_2684
PPRCHA0_2234
algA3
PPRCHA0_1062
PPRCHA0_1039
intergenic 1
PPRCHA0_0194
