# Supplementary material for: The insect- and plant-associated lifestyles of Pseudomonas protegens CHA0 are preserved following serial passage through insect larvae
Source: PLoS Pathog. 2026 Sep 21;22(9):e1014101. doi: 10.1371/journal.ppat.1014101 (PMC13592716; doi:10.1371/journal.ppat.1014101)
Supplement: Supplementary file 34 [file ppat.1014101.s037.pdf]

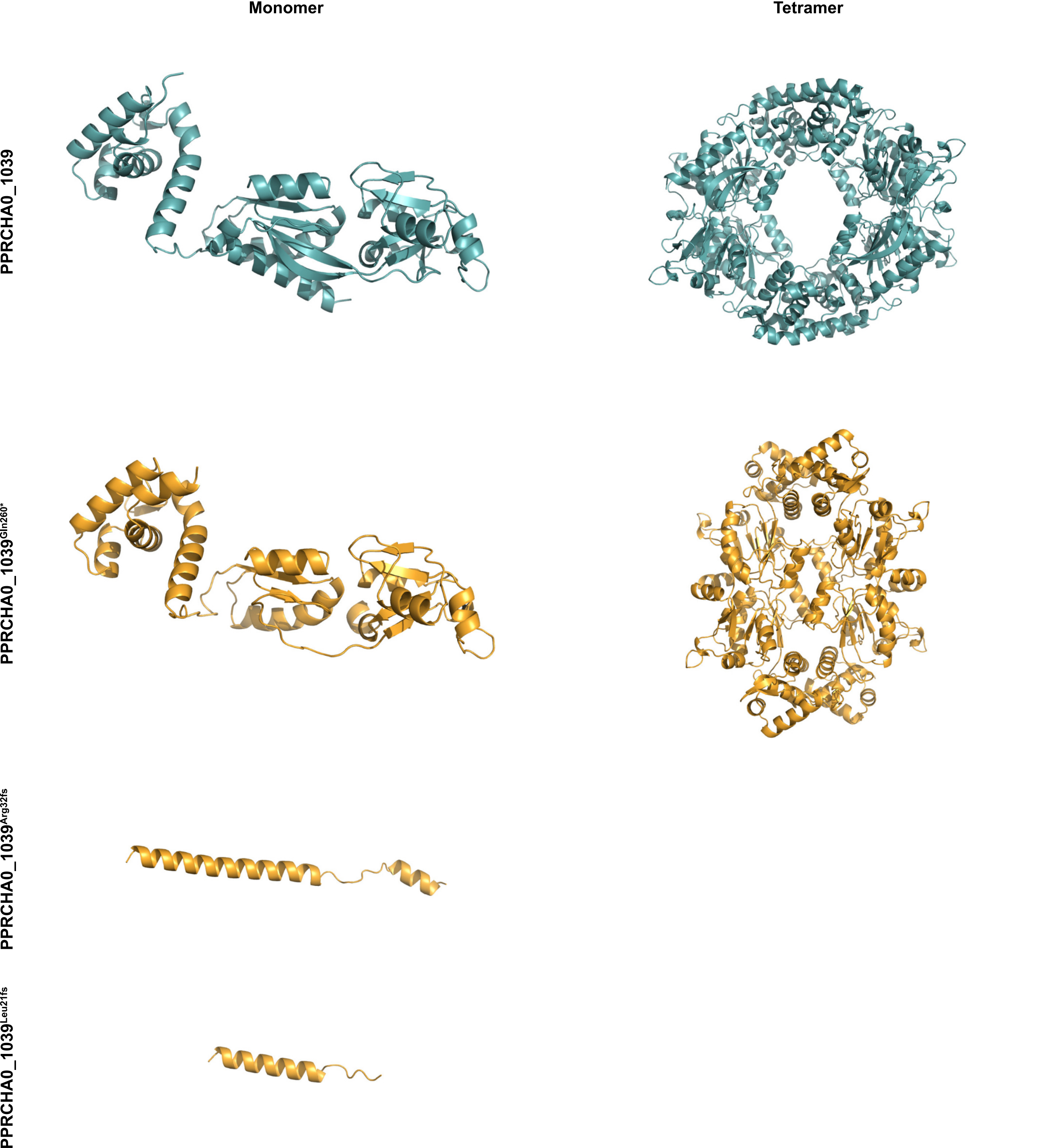
Monomer
Tetramer
PPRCHA0_1039
PPRCHA0_1039Gln260*
PPRCHA0_1039Arg32fs
PPRCHA0_1039Leu21fs
