# Supplementary material for: The insect- and plant-associated lifestyles of Pseudomonas protegens CHA0 are preserved following serial passage through insect larvae
Source: PLoS Pathog. 2026 Sep 21;22(9):e1014101. doi: 10.1371/journal.ppat.1014101 (PMC13592716; doi:10.1371/journal.ppat.1014101)
Supplement: Supplementary file 36 [file ppat.1014101.s039.pdf]

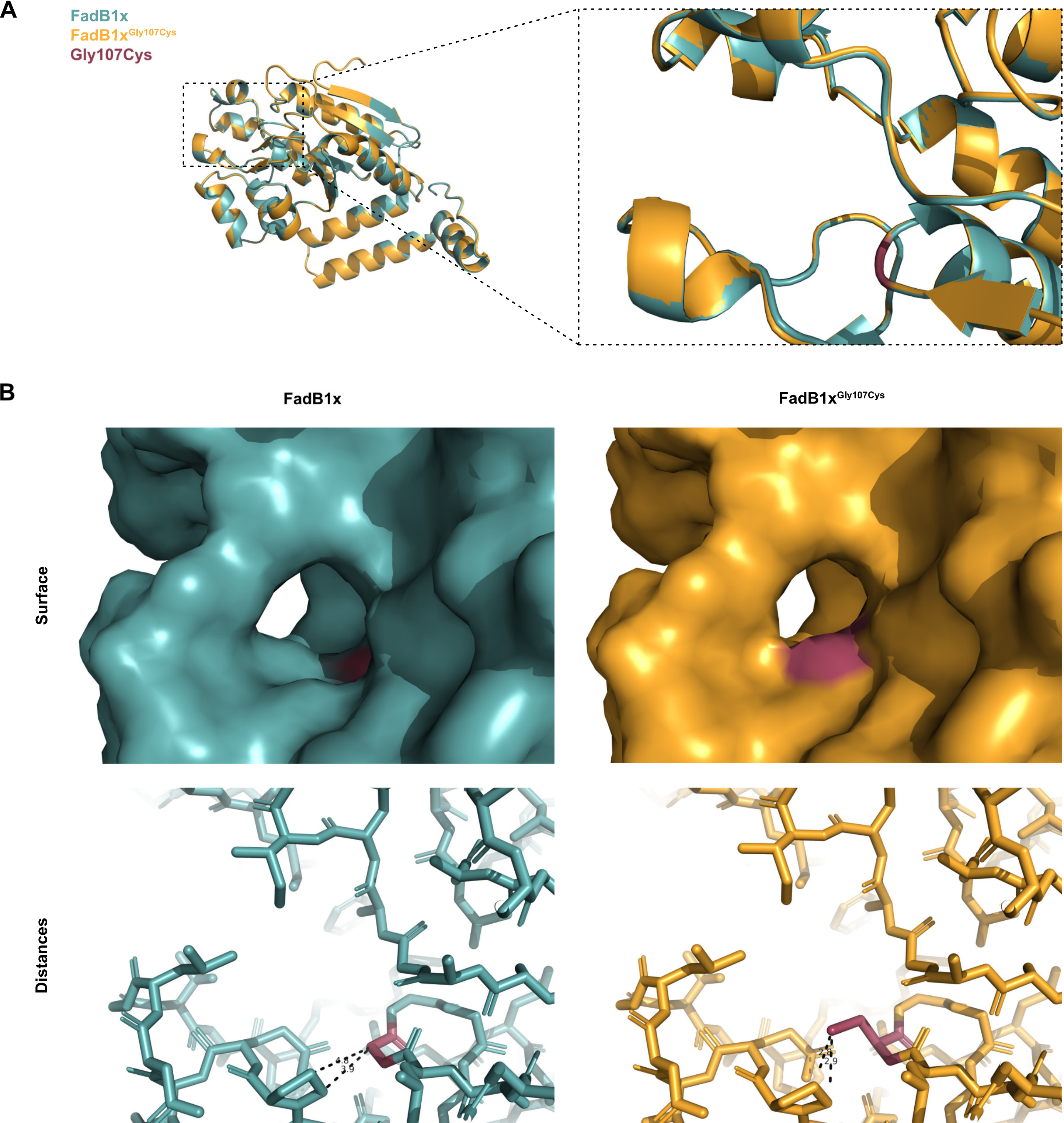
A
FadB1x
FadB1xGly107Cys
Gly107Cys
B
FadB1x
FadB1xGly107Cys
Surface
Distances
4.8
3.9
2.8
2.9
